# How Area Agencies on Aging Contribute to Social Connection for Older Adults in Rural America

**DOI:** 10.1093/geroni/igaa057.2092

**Published:** 2020-12-16

**Authors:** Claire Pendergrast

**Affiliations:** Syracuse University, Syracuse, New York, United States

## Abstract

Social ecological models of health identify intrapersonal, interpersonal, institutional, community, and policy-level contexts as social factors influencing individual and population health outcomes. However how institutions such as Area Agencies on Aging (AAA) shape rural older adults’ social networks and influence health is little explored. This research examines institutional influences of social networks for rural older adults, particularly the social connections resulting from their AAA services and programs. AAAs are local social service organizations that coordinate home- and community-based supports. Our 2020 case study of a rural AAA in upstate New York involved in-depth semi-structured interviews with AAA staff, volunteers and participants included key themes related to older adults’ social networks, social wellbeing, and physical and mental health. Our findings have both theoretical implications for rural community social structure as experienced by older adults, and practical implications to build AAA’s capacity to address social isolation for rural older adults. Part of a symposium sponsored by the Rural Aging Interest Group.

